# Mechanisms of INO80 remodeling

**DOI:** 10.1042/BST20260693

**Published:** 2026-10-29

**Authors:** Shagun Shukla, Blaine Bartholomew

**Affiliations:** 1 Department of Epigenetics and Molecular Carcinogenesis, The University of Texas MD Anderson Cancer Center, Houston, TX 77054, U.S.A.; 2 Feinberg School of Medicine, Northwestern University Chicago, IL 60611, U.S.A.

**Keywords:** chromatin, DNA damage, DNA replication and recombination, eukaryotic gene expression, nucleosome remodeling

## Abstract

The INO80 complex is an evolutionarily conserved ATP-dependent chromatin remodeler with central roles in transcription, DNA replication, and genome maintenance. INO80 is unusual among chromatin remodelers in its ability to space nucleosomes, position nucleosomes in a DNA-shape-dependent manner, and exchange H2A-H2B dimers. Recent structural and biochemical studies have provided new insight into how these diverse activities are generated and regulated. Here, we discuss evidence that INO80 remodeling is controlled by two distinct forms of DNA sensing. The Arp8 module senses extranucleosomal DNA length and regulates nucleosome spacing, whereas the mechanical properties of DNA within the nucleosome influence DNA translocation and sequence-dependent nucleosome positioning. We further discuss a model in which INO80′s ATPase activity initiates entry-side DNA unwrapping and movement, generating a DNA bulge that propagates across the histone-octamer surface. Conformational changes in the Arp5 module may couple ATPase-driven DNA translocation to these changes in histone-DNA interactions. Together, these findings provide a mechanistic framework for understanding how INO80 integrates linker-DNA sensing, DNA mechanics, and ATP-dependent remodeling to generate distinct chromatin structures.

## Introduction

Eukaryotic genomes are packaged into chromatin, where nucleosomes restrict DNA access and must be dynamically repositioned during transcription, replication, repair, and recombination. This repositioning is carried out by ATP-dependent chromatin remodelers: SWI/SNF, ISWI, CHD, and INO80, which couple ATP hydrolysis to nucleosome sliding and eviction [1–3]. The INO80 chromatin remodeler was first discovered in *Saccharomyces cerevisiae* and is distinguished from other remodelers by containing actin, actin-related proteins (ARPs), and RVB1/2 proteins that are AAA+ ATPases, in addition to the Ino80 catalytic subunit [4,5]. Later, other INO80-like complexes were found and together constitute the INO80 family of remodelers, which includes, in yeast, SWR1 [6–8] and, in higher eukaryotes, SRCAP [9,10] and EP400 (TIP60/p400) [9,11]. Instead of mobilizing nucleosomes like INO80, SWR1 and SRCAP deposit the histone variant H2A.Z and remove canonical H2A from nucleosomes without significant nucleosome movement [7,12]. The EP400 remodeler has the additional capacity to acetylate histones using the histone acetyltransferase TIP60 and removes or deposits H2A.Z depending on genomic context. In the INO80 family of chromatin remodelers, INO80 is the only one that beside histone exchange efficiently mobilizes nucleosomes and either spaces nucleosomes or positions them in a DNA-shape-dependent manner.

The present review covers recent findings on the mechanisms of INO80’s nucleosome spacing and DNA-shape-sensitive positioning of nucleosomes. For more information on the structural properties of INO80, we refer the reader to an earlier review [13]. Throughout the present review, we refer to the complex as INO80 and its catalytic subunit as Ino80.

## Structural organization and regulatory modules of INO80

The INO80 complex is organized around a central Snf2-family ATPase subunit, which serves as the overall assembly platform for the complex [14,15]. The helicase-SANT-associated (HSA) domain flanking the ATPase domain recruits actin (Act1), Arp4, and Arp8 to form the Arp8 module, which engages entry-side linker DNA and regulates remodeling activity [16,17]. The spacer insertion specifically interacts with the RuvBL1/RuvBL2 (Rvb1/Rvb2) AAA+ ATPase ring, which in turn helps organize the Ies2 and Arp5–Ies6 modules [15,18,19].

Cryo-EM studies revealed an elongated and asymmetric architecture in which the Rvb1/Rvb2 ring occupies one end of the complex, the ATPase motor engages nucleosomal DNA at superhelical location (SHL)−6, and the Arp8 module extends toward entry-side extranucleosomal DNA (Figure 1A) [15,20,21]. This arrangement positions the ATPase motor between two substrate-sensing systems: the Arp8 module, which monitors linker DNA, and the Arp5 module, which grips the nucleosome core. Arp5 engages nucleosomal DNA near SHL−2/−3 and contacts the proximal H2A–H2B interface, and Ies2 contacts the distal acidic patch. This pincer-like architecture couple linker-DNA sensing, histone-core engagement, and ATP-driven DNA translocation into productive remodeling [17,18,21].

**Figure 1:**
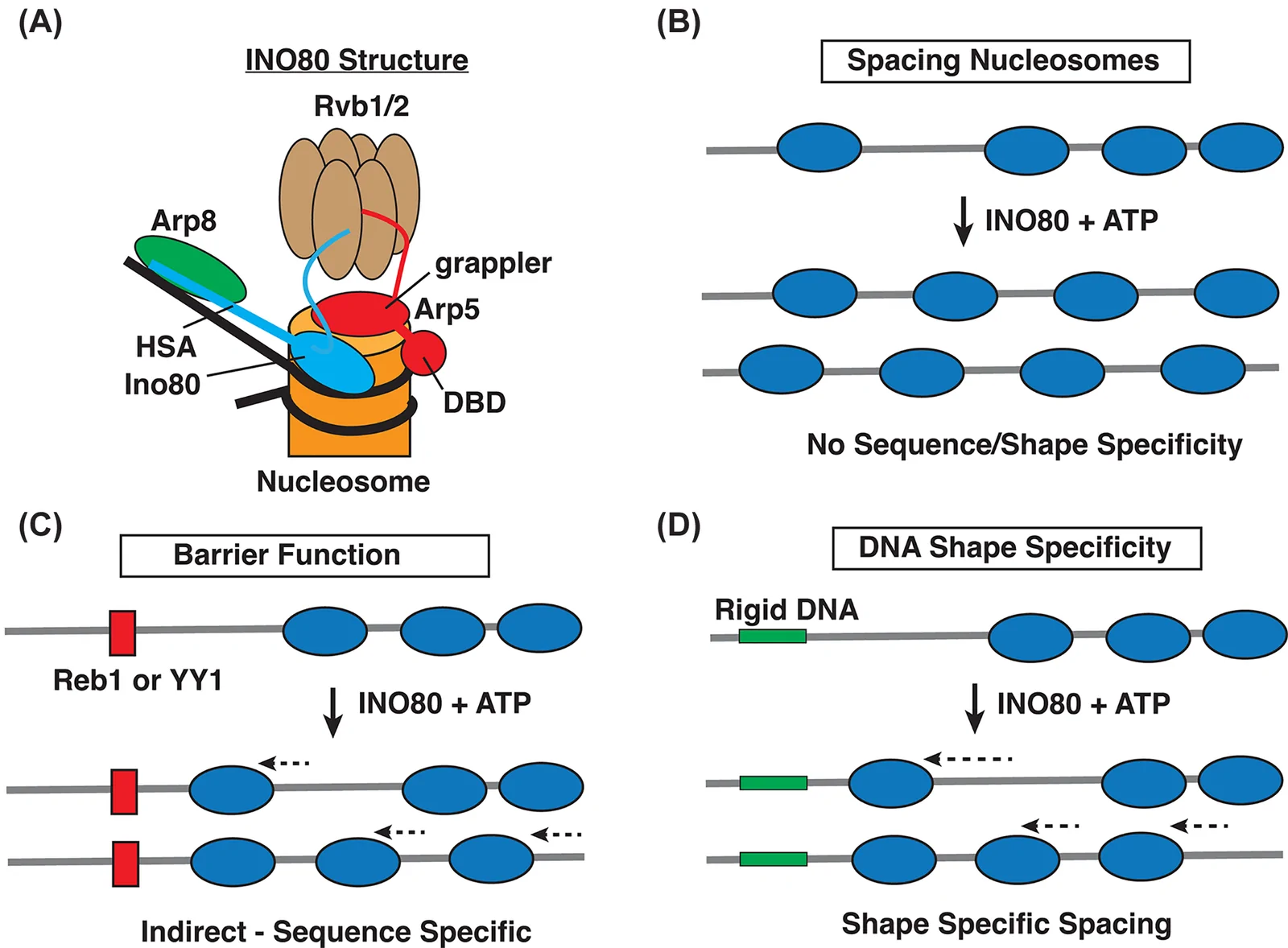
Organization and nucleosome sliding properties of INO80 (**A**) The different modules (Arp8 and Arp5) and subunits (Ino80 and Rvb1/2) of INO80 are shown and where they locate relative to the nucleosome and linker DNA. Different ways in which INO80 forms arrays of equally spaced nucleosomes are shown, which include in (**B**) DNA sequence-independent or -dependent manner with (**C**) DNA sequence-specific transcription factors or (**D**) DNA shape serving as a barrier.

## Nucleosome spacing activity of INO80

Starting 15 years ago, INO80 was discovered to move nucleosomes in an extranucleosomal DNA length-sensitive manner to create arrays of nucleosomes separated by an equal length of linker DNA (Figure 1B) [22]. The rate of nucleosome movement by INO80 was found to increase ∼100-fold when extranucleosomal DNA length is increased from 40 to 80 bp [19,23]. The Arp8 module consisting of Arp8, Arp4, actin, Taf14, and Ies4 subunits and the HSA domain is crucial for the DNA length-dependent regulation of INO80 and binds ∼40 bp of extranucleosomal DNA starting ∼20 bp from the nucleosome entry site [17,24]. Nucleosome movement by INO80 is inhibited by interfering with binding of the Arp8 module to DNA through mutating the HSA domain or truncating the N-terminus of Arp8 [24,25]. Loss of Arp8 module binding to linker DNA causes the Arp5/Ies6 module of INO80 to disengage from the histone octamer face, which is required for coupling ATP hydrolysis to nucleosome movement [24]. The Nhp10 subunit of *Saccharomyces cerevisiae* (*Sc*) INO80 was reported to be required for sensing linker DNA length like the Arp8 module, but in later genomic studies, deletion of Nhp10, mutation of its high-mobility group box, or removal of the entire Nhp10 was found not to affect nucleosome spacing, unlike mutations in the Arp8 module [19,25]. Nhp10 instead appears to have a more specialized role in targeting INO80 to DNA-damage sites through recognition of phosphorylated H2A (γ-H2AX) [26]. The ISW2 and ISW1a ATP-dependent remodelers also space nucleosomes like INO80 using a molecular ‘ruler’ that binds linker DNA and regulates their nucleosome remodeling activities [27–30].

The cryo-EM structure of the Arp8 module from *Chaetomium thermophilum* (*Ct*) and *Sc* has revealed that the subunits line up in the order of Arp8-actin-Arp4, with the HSA domain contacting all three [16,31]. These studies found the HSA domain and the N-terminus of Arp8 contact DNA, consistent with the earlier functional studies mentioned above. Mutation of lysines and arginines to glutamine in the α1 and α2 helices of the HSA domain and deletion of the N-terminus all reduced binding of the Arp8 module to the same extent [16]. Mutation in the α1 helix and truncation of the N-terminus similarly reduced remodeling, but mutations in the α2 helix essentially abolished remodeling [16]. While in most *Ct* INO80–nucleosome structures core INO80 and the Arp8 module are mobile with respect to each other, there was a subset with adequate resolution to visualize the allosteric communication between these two modules. This subset shows the Arp8 side of the module being on the side of DNA closest to the entry side of the nucleosome ∼20 bp away, and the HSA/post HSA forms one long continuous helix connecting the ATPase domain to the Arp8 module (Figure 1A) [16]. The post-HSA domain associates with the N-terminal lobe of the ATPase domain in the apo state and in the ADP•BeF_x_ bound state loses this interaction and becomes more flexible [16]. In the ADP•BeF_x_ bound state, the INO80 core and Arp8 modules appear to be more dynamically changing their orientation.

Recently cryo-EM studies with INO80 bound to nucleosomes with 40 bp of linker DNA revealed the Arp8 module is no longer bound to DNA but relocates and binds instead to the ATPase domain of INO80 [23]. Deletion of the Arp8 module from *Sc* INO80 was found to restore the mobilization of *Sc* nucleosomes with 40 bp of linker DNA. These data indicate INO80 stops moving nucleosomes when the length of linker DNA is ∼40 bp due to Arp8 switching its binding partner and inhibiting the ATP/translocation domain through direct interactions. In this study, they propose that the role of the Arp8 module is solely as an autoinhibitory domain. Key evidence for the Arp8 module being primarily an autoinhibitory domain comes from deletion or removal of Arp8 and mutations in Arp8’s N-terminal domain having a minimal impact on INO80 remodeling nucleosomes with 80 bp linker DNA, whereas these changes increased the rate of nucleosome movement ∼60-fold with nucleosomes that have 40 bp linker DNA [23]. These same changes in *Sc* INO80 with nucleosomes assembled with *Xenopus laevis* or human histones, however, severely blocked INO80 remodeling [16,24]. Since the nucleosome spacing and specific positioning of nucleosomes by INO80 do not depend on the species specificity of nucleosomes, it is likely that the Arp8 module having only an autoinhibiting function is not a universal property of INO80. These observations suggest that linker-DNA sensing and autoinhibition may represent different aspects of the same regulatory mechanism rather than mutually exclusive models. Binding of the Arp8 module to sufficiently long extranucleosomal DNA could relieve an inhibitory interaction with the ATPase domain, thereby coupling linker-DNA length to productive nucleosome translocation.

## Hexasomes are also remodeled by INO80

INO80 is less sensitive to flanking DNA length when remodeling hexasomes and implies INO80 remodeling might involve unwrapping DNA from the entry H2A-H2B dimer [32]. There remains uncertainty as to whether hexasomes is the preferred substrate for INO80, with one report using *Sc* INO80 reporting a 60-fold increase in the rate of remodeling [32], whereas two other studies using *Sc* and human INO80 have shown that nucleosomes are remodeled as efficiently as hexasomes [33,34].

When bound to hexasomes, INO80 interactions change dramatically with the ATPase domain shifting from SHL-6 to SHL-2, consistent with where DNA enters and binds [31,33]. In parallel, the Arp5 module also shifts and binds to the exposed H3-H4 tetramer and activates INO80 independent of H2A-H2B’s acid pocket [33]. These data show the Arp5 module can activate INO80 remodeling in two independent ways through interactions with the acid pocket or H3-H4 tetramer. The altered binding of INO80 seen with hexasomes may reflect intermediate states during INO80 remodeling of nucleosomes as the ATPase domain moves to a more internal nucleosome position.

## DNA shape specificity of INO80 and the Arp8 module

INO80 moves nucleosomes to unique genomic locations, many of which are proximal to gene promoters at the +1-nucleosome position and are distinct from nucleosome spacing that is not sequence specific (Figure 1C,D) [35,36]. Transcription factors like Reb1 in yeast or YY1 in mammals bind at gene promoters and function as barriers for INO80 remodeling [16,37] (Figure 1C). Fundamentally, the same mechanism is used as in nucleosome spacing and differs only in the type of barrier, whether an adjoining nucleosome or a sequence-specific transcription factor (Figure 1B). Most sites are, however, not positioned by TFs and instead are hardwired into the DNA sequence as seen *in vitro* using purified INO80 and yeast chromatin reconstituted with recombinant histones [38]. No distinct DNA motifs like poly dA-dT or GC-rich regions were found at these DNA regions that have been shown to block other remodelers [39–42].

Nucleosomes specifically positioned by INO80 are distinct from other nucleosomes assembled on the yeast genome with more negative propeller twist spanning ∼30 bp in the linker DNA region and more positive twist ∼20 bp on the entry side of nucleosomes [38]. Recent findings with DNA curtains found INO80 initially is more prone to bind DNA with higher propeller twist and to bind with longer lifetimes [43]. Higher negative propeller twist enhances base stacking with adjacent base pairs, often creating stiffly bent DNA as seen in A-tract DNA [44,45]. Cryo-EM found the Arp8 module binds to curved linker DNA and suggested this module might be responsible for the DNA shape specificity of INO80. Disrupting Arp8 binding by mutating the HSA domain, however, had a minor effect on nucleosome positioning, as seen in composite plots of MNase-seq data with nucleosomes shifting only 5-10 bp [25,38]. Complete loss of Arp8 binding blocks INO80 remodeling regardless of DNA shape and is universally required for INO80 moving nucleosomes regardless of the underlying DNA sequence. Other studies showed varying the physical properties of linker DNA did not significantly alter INO80 remodeling [35]. In contrast, DNA on the entry side of nucleosomes positioned by INO80 has a higher propensity for being less stiff and temporarily bent, which is discussed later in more detail.

The Arp8 module was recently shown to have an autoinhibitory function by switching from DNA to contacting and inhibiting the ATPase domain when linker DNA is shortened to 40 bp [23]. The autoinhibition function of the Arp8 module has been suggested to be the molecular basis for positioning +1 nucleosomes. This model, however, depends on specific positioning of the proximal nucleosome in the gene body and the necessity of blocking INO80 from moving +1-nucleosome-positioned nucleosomes into the nucleosome-free region, thus making it less tenable. The DNA shape model in contrast is not dependent on the positioning of adjacent nucleosomes or TFs and instead relies solely on the physical properties of DNA.

## The relationship between the mechanism of INO80 remodeling and INO80′s DNA shape specificity

Although INO80’s ATPase domain is structurally like other ATP-dependent chromatin remodelers, INO80 has a distinct mechanism for mobilizing nucleosomes. While the ATPase domain of other remodelers is bound to the SHL+/−2 position in nucleosomes, the ATPase domain of INO80 is bound at the SHL-6 position as shown by DNA footprinting, site-specific DNA crosslinking, and cryo-EM [20,21,46]. DNA footprinting shows DNA is not unwrapped when the ATPase domain of INO80 is bound, suggesting the unwrapped state observed by cryo-EM may represent a transient or selectively stabilized conformation rather than the predominant solution state.

Next, nucleosomal DNA 53 bp from the dyad on the side where DNA enters was found to be persistently unwrapped early in remodeling by tracking histone-DNA interactions between the H2A-H2B dimer [46]. These assays use a photo-reporter attached to residue 54 of H2B that photocrosslinks proximal DNA, making the crosslinked nucleotide more alkaline labile [47]. The site-specific DNA cleavage after photocrosslinking is used to temporally track DNA movement relative to the modified site on the histone octamer during remodeling. This method detected the initial unwrapping of DNA from the H2A-H2B dimer followed by DNA moving inside and rebinding 20 bp from its starting position on the H2A-H2B dimer (Figure 2A–D) [46]. These data are consistent with a later report of INO80 moving DNA 20 bp before reassessing DNA length using single-molecule FRET (fluorescence resonance energy transfer) [19].

**Figure 2:**
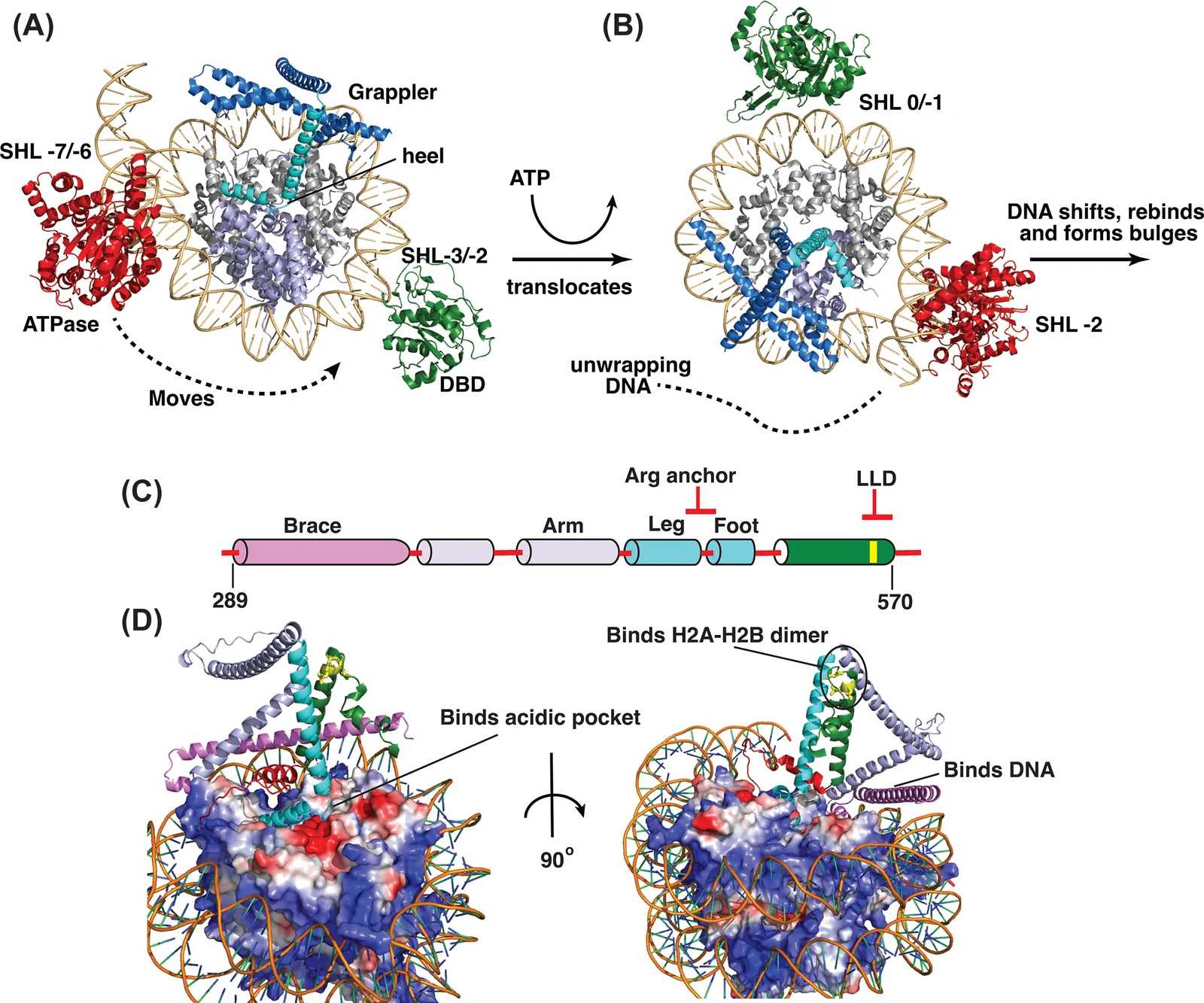
The bulge propagation model for INO80 mobilizing nucleosomes (**A,B**) The proposed movement of the grappler and DNA-binding domains (DBD) of Arp5 and the ATPase domain of INO80 during INO80 remodeling are shown, which are based on the cryo-EM structures of INO80 bound to nucleosomes (PDB: 8AV6) and hexasomes (PDB: 8007). During this proposed process DNA moves inside of nucleosomes, creating a DNA bulge that propagates around to the other side of the nucleosome. Dashed lines show the proposed movement. Shown are the foot and leg regions of Arp5 (cyan), the reminder of the grappler domain (blue), the H3-H4 tetramer (gray), and the H2A-H2B (light blue). (**C,D**) The structural components of the crossed conformation of the grappler domain are shown with the (**A**) legend and (**B**) two orientations of the model derived from cryo-EM data.

Nucleosomal DNA was also found to be unwrapped at other positions during INO80 remodeling. With limited ATP, kinetic DNA footprinting showed ∼30 bp of DNA bound to the H3-H4 tetramer being transiently unwrapped early during INO80 remodeling [48]. Mapping histone-DNA interactions on the side of nucleosomes where DNA exits during remodeling also revealed DNA being unwrapped from the histone octamer. When INO80 remodeling is abruptly stopped, the unwrapped DNA collapses back onto the histone octamer after shifting 30 bp of DNA out the exit side [49]. These data can be stitched together to infer that INO8O remodeling begins by unwrapping and shifting DNA further inside on the entry side of the nucleosome to form a DNA bulge on the histone octamer surface, and the DNA bulge moves around the nucleosome and ultimately exits out the other side. SWR1, a paralogous complex to INO80 with a similar three-dimensional structure and shared and paralogous subunits, also unwraps large stretches of nucleosomal DNA as seen using single-molecule FRET [50,51]. Although similar, these two complexes remodel nucleosomes differently, including where the ATPase domain engages nucleosomes, SHL-6 (INO80) versus SHL-2 (SWR1), and only INO80 mobilizes nucleosomes. The reason for their different mechanisms of nucleosome remodeling remains unclear, and how DNA translocation and unwrapping within nucleosomes produces markedly different outcomes.

601 nucleosomes were found to have one direction which INO80 remodels >20 times slower than the other [48]. Swapping different parts of the 601 sequence revealed that the slower remodeling depended on 36 bp of DNA on the entry side bound to the H2A-H2B dimer. This same 36 bp sequence was predicted to be more rigid than the rest of the 601 DNA using DNAcycP2, and this degree of rigidity is not recapitulated by any segment of the 36 bp region alone [48]. Partial segments of the 36 bp sequence placed on the entry side of 601 had only minor effects on blocking INO80 remodeling, indicating a higher level of rigidity was required for blocking INO80. Evidence for this not being unique to the 601 DNA came from first finding the 36 bp of DNA on the edge of nucleosomes positioned by INO80 on native yeast DNA is generally more rigid [48]. Representatives of these yeast DNAs when inserted into the entry side of 601 similarly block INO80 remodeling. The unusual propeller twist observed just inside of nucleosomes positioned by INO80 on yeast genomic DNA reflects similar characteristics to that observed for the 36 bp region in the 601 DNA. Together these data support the model of rigid DNA inside the entry side of nucleosomes blocking INO80 remodeling and accounting for INO80’s unusual DNA specificity.

Further evidence for the bulge propagation model came by tracking DNA movement and unwrapping on 601 nucleosomes, where the rigid DNA blocks INO80 remodeling. The initial unwrapping of DNA from the entry H2A-H2B occurs slightly faster; however, DNA movement inside followed by rebinding of DNA to the H2A-H2B dimer is blocked [48]. These data agree with rigid DNA interfering with the bending required for unwrapping DNA to shift its position relative to the histone octamer and rebinding. Not being able to form this initial bulge on the histone octamer surface due to rigid DNA also impedes DNA unwrapping at the surfaces of the H3-H4 tetramer and the exit side H2A-H2B [48]. These findings agree with rebinding of DNA on the entry side preceding DNA being unwrapped from the H3-H4 tetramer and exiting at the H2A-H2B surface on the opposite side, consistent with the bulge propagation model (Figure 2A,B).

## Arp5 module potential role in the DNA shape specificity of INO80

Arp5 is implicated in being involved at the bulge formation step due to its DBD binding at SHL-3 and proximity to the rigid DNA. The initial stages of INO80 remodeling can be modeled based on the cryo-EM structures of INO80 bound to nucleosomes and hexasomes with the ATPase domain moving from SHL-6 to SHL-2 and DNA progressively being more unwrapped from the nucleosome core like that shown in the biochemical studies mentioned previously (Figure 2A,B). This model highlights that as the ATPase domain moves around the nucleosome, there are corresponding changes with the DBD and grappler domain of the Arp5 module binding to nucleosomal DNA and the nucleosome core. DNA footprinting and site-specific histone crosslinking revealed the interactions of Arp5’s DBD and grappler domain at SHL-2 and near residue 80 of histone are reduced with rigid DNA at the entry side [48]. Kinetic DNA footprinting provides evidence for the DBD moving toward the dyad early in INO80 remodeling with additional protection at SHL-2 that appears to be coupled with DNA unwrapping off the tetramer. This approach demonstrated that rigid DNA blocks this additional protection and suggests it interferes with DBD movement [48]. Combined, these results indicate rigid DNA may perturb the initial binding of the DBD and grappler domains of Arp5 and block the initial movement of DBD.

## The Arp5 grappler domain is inherently flexible and mobile

The grappler domain of Arp5 was first shown by cryo-EM to adopt two distinct conformations that are referred to as parallel and crossed [21]. The crossed conformation appears to represent a remodeling competent state, while the role of the parallel conformation is yet to be determined [16]. One arm of the grappler domain in the crossed conformation binds alongside nucleosomal DNA, and its tip contacts the ATPase domain (Figure 2C,D, brace region). Mutation of the residues predicted from the structure to bind nucleosomal DNA strongly down-regulated the nucleosome sliding activity of INO80 [16]. In the crossed conformation there is a region called the foot and heel that positions three arginines at the acidic pocket of the nucleosome (Figure 2C,D). Changing these arginines to alanine down-regulated nucleosome sliding and disrupted grappler domain binding to the acidic pocket, as shown by histone-Arp5 crosslinking at residue 89 of histone H2A [33,49]. The structure of INO80 bound to hexasomes demonstrates the heel-foot region of the grappler domain can alternatively contact the α2-helix of histone H4 (Figure 2A,B) [31,33]. This interaction may not be reserved to only hexasome-bound INO80 because histone-Arp5 crosslinking shows the heel-foot region associated close to the α2-helix of histone H4 with nucleosome-bound INO80 [49]. This last finding suggests there may be other conformations or interactions of the grappler domain that are not readily detected by cryo-EM due to its dynamic nature.

There are functional aspects of Arp5 that appear to not require the arginine anchor even though it is vital for INO80 mobilizing nucleosomes, as shown by the Arp5 subunit being required but not the arginine anchor for INO80’s role in DNA replication stress and DNA repair [49]. Alanine scanning mutagenesis of Arp5 revealed Leu 567, Leu 569, and Asp 571 (LLD region) are required for INO80’s role in repair and replication [49]. The LLD region is at the tip of an alpha helix associated with the leg region of the grappler domain distal to the arginine anchor (Figure 2C,D). These mutations reduce the rate of nucleosome sliding, grappler domain binding proximal to the acidic pocket and α2 helix of H4, and preferentially impair INO80’s unwrapping of DNA on the entry side of nucleosomes [49]. Unexpectedly, the LLD region was shown to bind the H2A-H2B dimer using an *in vitro* binding assay with recombinant H2A-H2B dimers and a short peptide containing the LLD region [49]. Binding of the LLD peptide to H2A-H2B was blocked when Leu 567, Leu 569, and Asp 571 were changed to Ala and indicates its ability to bind H2A-H2B is likely important *in vivo* for INO80's role in DNA replication and repair. Histone crosslinking indicates the LLD region of Arp5 binds proximal to the acidic pocket region and suggests yet another mode of the grappler domain engaging the lateral face of the histone octamer [49]. Although these findings connect Arp5-H2A-H2B interactions with INO80 functions in replication and repair, whether the relevant *in vivo* activity is dimer exchange, nucleosome sliding, or another remodeling intermediate remains unresolved.

## Dimer exchange by INO80

These structural and biochemical assays suggest INO80 could mediate H2A/Z-H2B dimer exchange by displacing nucleosomal DNA from the H2A/Z-H2B dimer to weaken the interactions between the dimer and H4, thereby promoting its exchange for the H2A-H2B dimer. Yeast and human INO80 have been shown *in vivo* to have dimer exchange activity. Depletion of the catalytic subunit of INO80 blocks the removal of H2A.Z from DNA double-stranded breaks where H2A.Z is normally transiently deposited and functions like the histone chaperone ANP32E for H2A.Z removal [52]. In yeast there is a connection between H2A.Z and INO80 that is suggested by the substitution of four lysines in H2A.Z partially suppresses the sensitivity of *Ino80* mutants to replication stress and DNA damage [53]. In primed mouse embryonic stem cells, INO80 facilitates H2A.Z occupancy at bivalent promoters, which contrasts with other examples where H2A replaces H2A.Z [54].

It has been challenging and controversial to observe *in vitro* the dimer exchange activity of INO80 [53,55–57]. Single-molecule FRET experiments provided evidence for purified INO80 replacing H2A.Z for H2A being linked to DNA unwrapping at the entry side, which is coupled to DNA translocation and torsional strain near SHL-6 [46]. The difficulty of a robust *in vitro* dimer exchange assay for INO80 may be indicative of a missing factor or chaperone that might be required in combination with INO80. Further experiments are needed to better understand the mechanism and factors involved in INO80 H2A-H2B dimer exchange and its connection for mobilizing nucleosomes.

## Summary

Recent structural and biochemical studies suggest that INO80 integrates two distinct forms of DNA sensing to regulate nucleosome remodeling. The Arp8 module binds extranucleosomal DNA and regulates remodeling according to linker-DNA length, providing a mechanism for nucleosome spacing. INO80 also responds to the mechanical properties of DNA within the nucleosome. Remodeling begins with unwrapping and movement of entry-side DNA, followed by DNA unwrapping from the H3-H4 tetramer and exit-side H2A-H2B dimer. These observations support a model in which ATPase-driven DNA translocation generates a DNA bulge that propagates across the histone-octamer surface. Rigid entry-side DNA blocks these events, providing a potential mechanism for DNA-shape-dependent nucleosome positioning. Determining how these activities are coupled and the contribution of Arp5 conformational changes to sliding and histone exchange are important challenges for understanding the mechanism of INO80 remodeling. Most importantly, direct visualization of the proposed DNA-bulge intermediate and its movement around the histone octamer remains an important test. Addressing these questions will require integrating high-resolution structural approaches with kinetic, single-molecule, crosslinking, and solution-based measurements capable of resolving transient remodeling intermediates.

## Perspectives

- Importance of the field: ATP-dependent chromatin remodelers are essential for regulating nucleosome organization and DNA accessibility during transcription, replication, and DNA repair. INO80 is particularly important because it combines nucleosome spacing and positioning with histone-dimer exchange and responds to both extranucleosomal and nucleosomal DNA. Understanding how these activities are coordinated is therefore important for defining how chromatin structure is established and dynamically reorganized during genome regulation and maintenance.
- Current thinking: Recent structural and biochemical studies support a model in which INO80 integrates multiple forms of substrate sensing with ATP-dependent DNA translocation. The Arp8 module senses extranucleosomal DNA length and regulates nucleosome spacing with regulated autoinhibition, whereas the mechanical properties of DNA within the nucleosome influence productive DNA movement and nucleosome positioning. ATPase-driven DNA unwrapping on the entry side is proposed to generate a DNA bulge that propagates across the histone octamer surface, with conformational changes in Arp5 coupling DNA translocation to changes in histone-DNA interactions. Related disruption of H2A-H2B-DNA interactions may also contribute to histone-dimer exchange.
- Future directions: A major challenge will be to directly visualize the transient intermediates that connect ATP hydrolysis to DNA movement through the nucleosome. Determining how Arp8 communicates linker-DNA information to the ATPase-Arp5 machinery, whether the alternative Arp5 conformations represent sequential remodeling intermediates, and how DNA mechanics determine where nucleosome movement terminates will be particularly important. It will also be necessary to establish how a common remodeling mechanism is directed toward nucleosome sliding versus histone-dimer exchange and to determine the contribution of these activities to INO80 functions during transcription, DNA replication, and repair *in vivo*

## Open Access

Open access for this article was enabled by the participation of the University of Texas MD Anderson Cancer Center in an all-inclusive Read & Publish agreement with Portland Press and the Biochemical Society.

## Abbreviations

ARPs: actin-related proteins
*Ct*: *Chaetomium thermophilum*
DBD: DNA-binding domain
FRET: fluorescence resonance energy transfer
HSA: helicase -SANT associated
*Sc*: *Saccharomyces cerevisiae*
SHL: superhelical location
